# Кадровые проблемы эндокринологической службы и стратегии их решения

**DOI:** 10.14341/probl12853

**Published:** 2021-12-30

**Authors:** Е. А. Пигарова, С. Ю. Воротникова

**Affiliations:** Национальный медицинский исследовательский центр эндокринологии; Национальный медицинский исследовательский центр эндокринологии

**Keywords:** кадровое обеспечение, эндокринология, эндокринологическая служба, организация здравоохранения

## Abstract

Эффективность системы здравоохранения во многом определяется кадровым составом и квалификацией врачей. Укомплектование персоналом медицинских организаций, в частности первичного звена, — важнейший аспект качества и доступности медицинской помощи, а также экономики в целом. Несмотря на имеющиеся кадровые проблемы эндокринологической службы, усиление роли государства в решении данного вопроса в виде долгосрочного стратегического планирования и повышения квалификации специалистов, увеличения объемов целевой подготовки врачей, а также их материальной и социальной поддержки позволяет надеяться на переход положительной динамики в уверенное разрешение кадрового кризиса.

## Abstract (en)

The effectiveness of the healthcare system is largely determined by the staffing and qualifications of doctors. The human resources of the health care system is a critical aspect of the quality and accessibility of healthcare and the economy as a whole. Despite the existing staffing problems of the endocrinological service, the strengthening of the role of the state in solving this issue in the form of long-term planning of staffing, approaches to targeted training and distribution of specialists, as well as their material and social support, allows us to hope for the transition of positive dynamics into a confident resolution of the personnel crisis.

Проблемы кадрового обеспечения, особенно в сфере здравоохранения, стали намного сложнее. Укомплектование персоналом системы здравоохранения — важнейший аспект качества и доступности медицинской помощи, а также экономики в целом.

В докладе Всемирной организации здравоохранения (ВОЗ) на Третьем глобальном форуме по кадровым ресурсам здравоохранения («A Universal Truth: No health without a workforce» = «Всеобщая реальность: без трудовых ресурсов нет здоровья»), прошедшем в ноябре 2013 г., дан прогноз, что в 2035 г. в мире будет не хватать 12,9 млн работников здравоохранения, что практически в 2 раза выше, чем установленный дефицит в 2013 г. В докладе названы основные причины кадрового дефицита работников здравоохранения (ВОЗ): старение и их выход на пенсию, переход медицинских работников на более высокооплачиваемую работу и отсутствие специалистов, которые могли бы прийти на освободившиеся места, недостаточное число молодых людей, желающих получить или уже получивших профессию врача, рост численности населения мира, подвергающегося рискам со стороны неинфекционных заболеваний, что приводит к большей потребности в медицинской помощи, а также внутренняя и международная миграция работников здравоохранения. В настоящее время все эти причины являются высокоактуальными, и на их решение направлены умы и ресурсы большинства стран мира, как с высокими экономическими индексами, так и развивающихся.

Структура кадрового дефицита в различных странах представлена по-разному. Это может быть изолированный дефицит кадров в первичном звене здравоохранения (терапевты, педиатры, врачи общей практики), врачей-специалистов (например, врачей-эндокринологов) или среднего медицинского персонала (медицинские сестры, фельдшеры). При изолированном дефиците существуют возможности перераспределения нагрузки между этими звеньями с поддержкой направления кадрового дефицита. Если же дефицит кадров затрагивает все звенья, то решить вопрос с перераспределением невозможно без подготовки новых кадров и их удержания на местах, это накладывает особую ответственность на органы исполнительной власти в области здравоохранения по своевременному планированию кадрового обеспечения и мерах их поддержки.

Пандемия COVID-19 стала дополнительным мощным фактором проверки системы здравоохранения на прочность. Многие эндокринологические стационары и врачи были перепрофилированы для лечения пациентов с новой коронавирусной инфекцией, что привело к ограничению доступности плановой медицинской помощи. Произошел также одновременный рост различных эндокринологических нарушений у пациентов с COVID-19 как вследствие самого заболевания, так и его лечения, что, безусловно, обострило проблемы кадрового дефицита эндокринологической службы.

В 2021 г., по данным Росстата, дефицит врачей по специальности «эндокринология» составил по стране в среднем 24% (1954 из 8071) и по специальности «детская эндокринология» — 38% (620 из 1651).

ФГБУ «НМИЦ эндокринологии» Минздрава России по заданию Минздрава России на регулярной основе проводит выездные мероприятия по аудиту эндокринологической службы в субъектах Российской Федерации. В ходе этих мероприятий рекомендации по улучшению кадровой ситуации в медицинских организациях регионов обсуждаются с органами исполнительной власти в сфере охраны здоровья субъектов Российской Федерации.

Основные кадровые проблемы в здравоохранении по данным анализа выездов — это не только абсолютный дефицит медицинских кадров, но и диспропорции в их составе. Выявлены невысокий профессиональный уровень различных звеньев медицинского персонала и в целом низкая мотивация специалистов, что связано с неэффективным использованием рабочего времени и неудовлетворительными условиями труда, а также слабой материально-технической базой медицинских учреждений.

Большой вклад в ликвидацию компетентностных пробелов эндокринологов и детских эндокринологов вносит ФГБУ «НМИЦ эндокринологии» Минздрава как учреждение, оказывающее организационно-методическое сопровождение деятельности медицинских организаций субъектов Российской Федерации по профилю. В 2020 г. на основании данных всероссийского анкетирования сотрудниками центра выделены приоритетные тематики для разработки и реализации образовательных программ повышения квалификации, включающие вопросы ведения пациентов с нейроэндокринными заболеваниями, водно-электролитными нарушениями, гиперпаратиреозом и др. В течение 2 лет в центре прошли обучение более 1000 специалистов из 74 регионов России и ближнего зарубежья.

С января 2021 г. начал функционировать информационно-образовательный портал «Эндосфера» (edu.endocrincentr.ru), который объединяет функции основной платформы проведения дистанционного обучения, агрегатора аккредитационных мероприятий, а также является транслятором обновлений позиций ведущих профессиональных сообществ, публикаций в медицинских журналах, профессиональных новостей и мнений экспертов.

Направления по решению кадрового вопроса эндокринологической службы, несомненно, заключаются в реорганизации образовательной стратегии в области эндокринологии, расчете и планировании ликвидации кадрового дефицита, аттестации профильных кафедр и их профессорско-преподавательского состава, стандартизации учебного процесса подготовки эндокринологов с внедрением современных клинических рекомендаций и новых технологий диагностики и лечения, обучении практическим и коммуникативным навыкам, формировании клинического мышления, в том числе в симулированных условиях.

За последние 3 года отмечается положительная динамика в кадровом составе эндокринологической службы, что связано как с увеличением суммарного количества бюджетных мест, так и с внедрением целевого обучения и повышением доли целевых мест для обучения по программам подготовки кадров высшей квалификации в ординатуре в общем объеме контрольных цифр приема по профилю «эндокринология» с 67 до 83%, «детская эндокринология» — с 44 до 96%. Важный фактор — повышение мотивации и вовлеченности главных внештатных специалистов, региональных органов исполнительной власти в области здравоохранения и вузов в формировании долгосрочного плана по подготовке кадров по профилю.

Учитывая высокую потребность практического здравоохранения в эндокринологах, а также интенсивность труда данной категории врачей, целесообразным представляется перенос части функциональных обязанностей медицинских специалистов с высшим образованием на квалифицированный сестринский персонал, что наиболее актуально в области терапевтического обучения пациентов с сахарным диабетом. В связи с этим сотрудниками ФГБУ «НМИЦ эндокринологии» Минздрава России в 2021 г. разработана дополнительная профессиональная образовательная программа повышения квалификации медицинских сестер «Ведение школы для больных сахарным диабетом». Актуальность данной программы (срок обучения 72 академических часа) обусловлена необходимостью подготовки специалистов среднего медицинского звена, способных на современном уровне проводить обучение пациентов с сахарным диабетом, а также родителей детей и иных лиц, осуществляющих уход за пациентами с сахарным диабетом. Обучение является неотъемлемой частью комплекса терапевтических мероприятий при сахарном диабете, крайне необходимо для его эффективного контроля и должно продолжаться на всем протяжении заболевания.

Раннее выявление эндокринологических заболеваний — ключевой фактор для дальнейшего успешного лечения и недопущения тяжелых осложнений, в связи с чем как крайне важное оценено внедрение новой компетенции «эндокринологической настороженности» в первичное звено здравоохранения. Это было реализовано в виде разработанного электронного образовательного курса, представленного для изучения на Портале непрерывного медицинского и фармацевтического образования Минздрава России. Обучение на данном курсе прошли более 50 000 специалистов не эндокринологов, что имеет крайне важное значение при перераспределении медицинских кадровых ресурсов в условиях пандемии для сохранения качества оказания медицинской помощи пациентам с эндокринной патологией.

Таким образом, несмотря на имеющиеся кадровые проблемы эндокринологической службы, усиление роли государства в решении данного вопроса в виде долгосрочного планирования кадрового обеспечения, подходов целевого обучения и распределения специалистов, а также их материальной и социальной поддержки позволяет надеяться на переход положительной динамики в уверенное разрешение кадрового кризиса.

## ДОПОЛНИТЕЛЬНАЯ ИНФОРМАЦИЯ

Источники финансирования. Работа выполнена по инициативе авторов без привлечения финансирования.

Конфликт интересов. Авторы декларируют отсутствие явных и потенциальных конфликтов интересов, связанных с содержанием настоящей статьи.

Участие авторов. Все авторы одобрили финальную версию статьи перед публикацией, выразили согласие нести ответственность за все аспекты работы, подразумевающую надлежащее изучение и решение вопросов, связанных с точностью или добросовестностью любой части работы.

